# Tuberculosis

**Published:** 1895-06

**Authors:** 


THE Royal Commission on tuberculosis, appointed in 1890, has recently made its report to Parliament, specially as to the danger from animal foods.

They consider the identity of human aud bovine tuberculosis as established beyond doubt.

That tuberculous food eaten reproduced the disease.

That the method of the removal of the meat, the results of inoculation and of feeding, forces the conclusion that when meat is infective it commonly acquires its properties by being accidentally contaminated with tuberculous material during its removal from the carcass.

That milk from cows with tuberculous udders was extremely virulent when fed to test-animals, and, further, that butter obtained from milk from such an animal contained material actively injurious to test-animals. They advise the prompt removal from every dairy of every tuberculous cow with diseased udders.

That the influence of cooking was often overrated through conclusions that the temperature reached had been high enough to destroy all germs, when deep centres of the meat had not been reached with sufficient heat to destroy.

That all milk should be boiled when suspected.

